# Long-term immune responses and comparative effectiveness of one or two doses of 7-valent pneumococcal conjugate vaccine (PCV7) in HIV-positive adults in the era of combination antiretroviral therapy

**DOI:** 10.7448/IAS.19.1.20631

**Published:** 2016-01-29

**Authors:** Aristine Cheng, Sui-Yuan Chang, Mao-Song Tsai, Yi-Ching Su, Wen-Chun Liu, Hsin-Yun Sun, Chien-Ching Hung

**Affiliations:** 1 Department of Internal Medicine, National Taiwan University Hospital Hsin-Chu Branch, Hsin-Chu, Taiwan; 2 Department of Clinical Laboratory Sciences and Medical Biotechnology, National Taiwan University College of Medicine, Taipei, Taiwan; 3 Department of Laboratory Medicine, National Taiwan University Hospital and National Taiwan University College of Medicine, Taipei, Taiwan; 4 Department of Internal Medicine, Far Eastern Memorial Hospital, New Taipei City, Taiwan; 5 Department of Internal Medicine, National Taiwan University Hospital and National Taiwan University College of Medicine, Taipei, Taiwan; 6 Department of Medical Research, China Medical University Hospital, Taichung, Taiwan; 7 China Medical University, Taichung, Taiwan

**Keywords:** serological response, anti-capsular antibody, immunogenicity, *Streptococcus pneumoniae*, invasive pneumococcal disease

## Abstract

**Introduction:**

HIV infection impairs maintenance of immunological memory, yet few studies of HIV-positive adults receiving 7-valent pneumococcal conjugate vaccine (PCV7) have followed them beyond the first year. We determined and compared the durability of serological responses and the clinical outcomes of HIV-positive adults annually for five years following vaccination with one or two doses of PCV7.

**Methods:**

In this non-randomized clinical trial, 221 pneumococcal vaccine-naïve HIV-positive adults receiving one (*n*=109) or two doses four weeks apart (*n*=112) of PCV7 between 2008 and 2010 were longitudinally followed for evaluation of significant serological response and for episodes of pneumonia and invasive pneumococcal disease.

**Results:**

At the time of vaccination, the two groups were well matched for age, risk factors, combination antiretroviral therapy (cART) coverage, CD4 count and plasma HIV RNA load (PVL). At the end of five years, the CD4 counts for the one- and two-dose groups had increased from 407 and 406 to 550 and 592 cells/µL, respectively, and 82.4 and 81.6% of the participants had fully suppressed PVL. Significant immune responses to ≥2 serotypes persisted for 67.9 vs 78.6%, 64.2 vs 71.4%, 66.1 vs 71.4%, 57.8 vs 69.6% in the second, third, fourth and fifth years after one and two doses of PCV7 in the intention-to-treat analysis, respectively. In multivariate analysis, immunization with two doses of PCV7 (odds ratio (OR) 1.71, 95% confidence interval (CI) 1.10 to 2.65, *p*=0.016), concurrent cART (OR 2.16, 95% CI 1.16 to 4.00, *p*=0.015) and CD4 proliferation (OR 1.12, 95% CI 1.01 to 1.27, *p*=0.031) were predictive of persistent serological responses in the fifth year. Only one patient in the one-dose group had documented pneumococcal pneumonia (non-bacteraemic) and none had invasive pneumococcal disease in the 6.5 years of follow-up.

**Conclusions:**

One or two doses of PCV7 achieve durable seroprotective responses in HIV-treated participants; however, two doses may be more robust than one dose in a larger study population or in real-world populations with less cART coverage.

## Introduction

Adults infected with the human immunodeficiency virus (HIV) are at significantly higher risk of invasive and recurrent pneumococcal infections despite combination antiretroviral therapy (cART) [1–3]. This risk may be related to HIV-related accelerated senescence of immune repertoire and loss of memory B cells prior to viral suppression and the relative dysregulation of the reconstituted but incompletely restored immune system following antiretroviral therapy [4–8]. Consequently most authorities, including the Advisory Committee on Immunization Practices of the US Centers for Disease Control and Prevention, the World Health Organization (WHO), the European AIDS Clinical Society and the British HIV Association, recommend pneumococcal vaccination for all HIV-positive adults regardless of immune status [9–13].

Strategies to optimize vaccine efficacy and effectiveness at the individual and public health levels vary from country to country. However, of the two different vaccines that have been developed (pneumococcal polysaccharide vaccine (PPV) and pneumococcal conjugate vaccine (PCV)), current guidelines recommend the latter, which elicits a T-cell-dependent response and memory B and T cells, as the initial vaccine for HIV-positive adults and children [10,11,14,15]. The 23-valent PPV may be administered subsequently to broaden serotype coverage [16]. Given the improved survival of HIV-positive persons on cART, when and how many doses should be administered as revaccination over the lifetime of HIV-positive persons following priming with vaccination with PCVs become important clinical issues [9].

Unfortunately, there is a dearth of data on the long-term immunogenicity of PCVs in HIV-positive adults, although we know that following PPV antibody concentrations drop below the cutoff values for most serotypes after five years; hence revaccination after five years is recommended [17–21]. Whether the same paradigm applies following PCV remains unanswered. We previously showed that HIV-positive adults on cART who received two doses of 7-valent PCV (PCV7) achieved better serological responses than those who received one dose during and at the end of 48 weeks of follow-up [22]. Here we followed this cohort longitudinally and investigated the durability and superiority of two doses over one dose of PCV7 during the five consecutive years of follow-up.

## Methods

### Study population and setting

HIV-positive adults aged ≥20 years who had no history of pneumococcal vaccination were recruited from infectious disease clinics at the National Taiwan University Hospital, the largest designated hospital for inpatient and outpatient HIV care in Taiwan, from October 2008 to June 2010. HIV infection was confirmed by Western blot. Participants with the following conditions were excluded: current pregnancy, use of immunomodulating agents within the past three months or use of cytoreductive chemotherapy within the last six months [22]. The study was approved by the Research Ethics Committee of the hospital and the participants gave written informed consent.

In Taiwan, HIV-positive patients have free access to HIV care that includes cART and monitoring of CD4 cell counts, plasma HIV RNA load (PVL) and biochemistry following the local HIV treatment guidelines. CART was defined as the combination of at least three antiretroviral agents that contained two nucleoside reverse-transcriptase inhibitors plus boosted or unboosted protease inhibitors or one non-nucleoside reverse-transcriptase inhibitor or integrase inhibitor or alternatively three nucleoside reverse-transcriptase inhibitors.

### Vaccine administration

All eligible participants were consecutively enrolled to receive one or two doses of vaccine four weeks apart administered by study nurses via intramuscular deltoid injections. This dosing schedule was used in the only efficacy trial of PCV7 in HIV-positive adults as well as earlier PCV7 immunogenicity trials [23,24]. Each 0.5-ml dose of PCV7 vaccine (Prevenar/Prevnar^®^, Wyeth-Lederle, New York, USA) contained 2 µg of capsular polysaccharide from each of six serotypes (4, 9V, 14, 18C, 19F and 23F) and 4 µg of capsular polysaccharide from serotype 6B, linked to 20 to 25 µg of CRM_197_. After vaccination, participants were prospectively followed and blood samples were collected every 12 weeks during the first 48-week follow-up period and annually for the subsequent four years of follow-up. Subjects in the two groups selected for final analysis were matched by CD4 count and PVL at vaccination.

### Laboratory investigations

PVL was quantified using the Cobas Amplicor HIV-1 Monitor test (Cobas Amplicor version 1.5, Roche Diagnostics, Indianapolis, IN, USA) with a lower detection limit of 20 copies/mL, and CD4 count was determined using FACFlow (BD FACS Calibur, Becton Dickinson, San Jose, CA, USA). The CD4 counts and PVL were monitored one month after initiation of cART in antiretroviral-naïve participants or after a change of regimen due to virological failure and every three to six months thereafter according to the local HIV treatment guidelines.

### Determinations of anti-capsular antibody levels

Serum samples were separated from clotted blood samples by centrifugation and stored at −70°C. Anti-capsular antibody concentrations to the four most prevalent pneumococcal serotypes (serotypes 6B, 14, 23F and 19F) in Taiwan [25] were determined in serially collected blood specimens using ELISA as previously described [26]. The antibody responses between the second and fifth years were determined using the new human pneumococcal standard reference serum, 007sp, and dual adsorption with cell-wall polysaccharide and pneumococcal polysaccharide 22F as specified in the WHO standard [27]. In previous years (4-week to 48-week ELISA responses), the concentration of immunoglobulin (IgG) used the original WHO-approved reference standard 89F, which did not specify the use of pneumococcal polysaccharide 22F [28]. However, since the reference standard 89F was exhausted and bridged to the 007sp serum, we used the new standard reference serum with dual adsorption. Comparison of the two methods revealed similar results for methods, especially at antibody levels of >1 µg/mL [29].

### Primary and secondary end points

The primary end point of the study was durable significant antibody responses, defined as a twofold or greater increase in specific IgG against two or more serotypes after five years [17,22,24]. Serological secondary end points included the geometric mean titres (GMTs), proportion of participants with sustained specific IgG concentrations above ≥1 µg/mL and the proportion of participants with persistent seroprotective responses, defined as a twofold or greater increase plus titres ≥1 µg/mL to at least two of four serotypes studied [24,30]. Antibody response rates were estimated by both intention-to-treat (ITT) analysis, in which participants with missing data were considered non-responders, and per-protocol (PP) analysis, in which participants with missing data were excluded from analysis. Clinical secondary end points included pneumonia, pneumococcal pneumonia and invasive pneumococcal disease (IPD) episodes. Pneumonia was defined by clinical presentation and radiographic findings consistent with community-acquired pneumonia. A diagnosis of probable pneumococcal pneumonia was made if a sputum smear with a Quality score of 3 yielded Gram-positive cocci in chains in the absence of another identifiable aetiology; the diagnosis was confirmed if, additionally, the sputum culture yielded *Streptococcus pneumoniae* or if the urinary pneumococcal antigen test was positive [31]. IPD was defined by the isolation of *S. pneumoniae* from a normally sterile site (i.e. blood, cerebrospinal or pleural fluid) [31]. The laboratory researchers who quantified the antibody responses were blinded to the identity, clinical and vaccination status of the participants. The radiologists who reported the radiographic findings were also blinded to the vaccination status of the participants.

### Statistical analyses

The analyses were conducted using the statistical package SAS 9.4 (SAS Institute Inc., Cary, NC, USA). Chi-square tests or, if necessary, Fisher's exact tests were used for categorical variables. Student's *t*-tests and Mann-Whitney U tests were used for numerical variables. Since observations were made over time periods, generalized estimating equations (GEEs) accounting for the interdependence among observations were used to compare mean response rates to different PCV doses, with adjustments made for time-updated variables including the patient's age at time of vaccination; at the second, third, fourth and fifth years; the CD4 counts at baseline, at nadir and at the second, third, fourth and fifth years (in increments of 100 cells/µL); PVL at baseline, peak PVL and suppressed PVL <20 copies/mL at the second, third, fourth and fifth years; co-infection with hepatitis B and hepatitis C virus (HCV) as defined by the presence of hepatitis B surface antigen (HBsAg) and anti-HCV antibody, respectively, at time of vaccination; and receipt of cART at time of vaccination and at the second, third, fourth and fifth years. A stepwise model comparison and selection were used to determine the final model of multiple variable analysis [32]. We used the SAS PROC GENMOD procedure to fit the GEE models. Odds ratios for each prognostic factor and 95% confidence intervals (CIs) were also calculated. All statistical tests were two-tailed, and *p* values <0.05 were considered significant.

## Results

**Figure 1:**
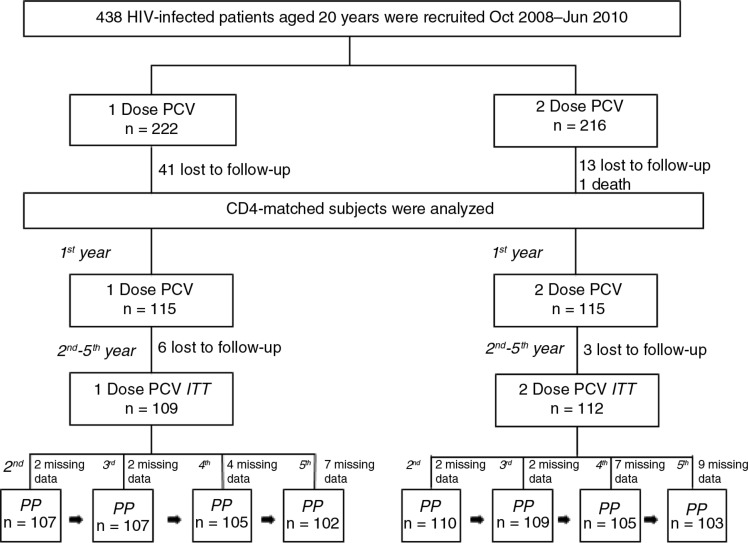
Study flow of HIV-positive adult participants receiving one or two doses of 7-valent pneumococcal conjugate vaccine followed for five consecutive years.

The study flow is shown in Figure 1. Serological responses beyond the first year were evaluated in 221 participants by ITT analysis, 112 of whom had received two doses of PCV7 and 109 one dose only. Their baseline clinical characteristics are shown in Table 1. The cohort comprised mainly male adults who had acquired HIV via sexual intercourse. At the time of vaccination, over 70% of the cohort were receiving cART but less than half had undetectable PVL. After five years of follow-up, the percentages of participants receiving cART and achieving undetectable PVL had increased to above 90% and 80%, respectively. The one- and two-dose groups were well matched for age, cART coverage, CD4 count and co-morbidities both at baseline and at the end of five years of follow-up. With the exception of the peak PVL, which was higher in the two-dose group compared to the one-dose group (5.8 log_10_ copies/mL vs 4.9 log_10_ copies/mL, *p*=0.025), the PVL at time of vaccination and at the end of follow-up were similar between groups.

**Table 1:** Characteristics of HIV-positive adults receiving primary vaccination with one or two doses of PCV7 at baseline and at the end of the five years of follow-up

	One dose (*n*=109)	Two doses (*n*=112)	*p*
Age, mean (SD), years	35.8 (10.1)	36.1 (10.8)	0.789
Male, *n* (%)	104 (95.4)	108 (96.4)	0.746
Risk factor, *n* (%)			0.520
Homosexual/bisexual male	93 (86.1)	87 (80.6)	
Heterosexual	13 (12.0)	17 (15.7)	
Injecting drug user	2 (1.9)	4 (3.7)	
Treatment status, *n* (%)			
On cART at baseline	77 (70.6)	81 (72.3)	0.782
On cART at end of five years	94 (92.2)	95 (92.2)	0.984
CD4 lymphocyte count, cells/µL, median (IQR)			
Nadir CD4	240 (79 to 387)	229 (51 to 450)	0.737
Baseline CD4	407 (244 to 583)	446 (252 to 591)	0.543
< 200, *n* (%)	13 (11.9)	15 (13.4)	0.743
200 to 349, *n* (%)	25 (22.9)	26 (23.2)	0.961
350 to 499, *n* (%)	31 (28.4)	26 (23.2)	0.375
≥ 500, *n* (%)	40 (36.7)	45 (40.2)	0.595
End of five-year CD4	550 (426 to 735)	592 (433 to 749)	0.476
< 200, *n* (%)	6 (5.6)	11 (10.0)	0.229
200 to 349, *n* (%)	21 (19.6)	14 (12.7)	0.167
350 to 499, *n* (%)	32 (29.9)	26 (23.6)	0.297
≥ 500, *n* (%)	46 (43.0)	59 (53.6)	0.117
Plasma HIV RNA load (PVL), log_10_ copies/mL			
Peak PVL, median (IQR)	4.9 (4.3 to 5.4)	5.8 (4.4 to 5.6)	0.025
Baseline PVL, median (IQR)	2.2 (1.6 to 4.0)	1.7 (1.6 to 3.9)	0.314
End of five-year PVL, median (IQR)	1.3 (1.3 to 1.3)	1.3 (1.3 to 1.3)	0.974
Undetectable PVL at baseline, *n* (%)	48 (44.0)	52 (46.4)	0.721
Undetectable PVL at five years, *n* (%)	84 (82.4)	84 (81.6)	0.882
Co-morbidities prevaccination, *n* (%)			
Chronic HBV co-infection	20 (19.0)	20 (17.9)	0.821
Chronic HCV co-infection	6 (5.6)	5 (4.5)	0.710
Isolated anti-HBc	48 (47.5)	45 (42.5)	0.463
Chronic pulmonary disease	1 (0.9)	6 (5.4)	0.119
Congestive heart failure	1 (0.9)	3 (2.7)	0.622
Diabetes mellitus	4 (3.7)	2 (1.8)	0.441

cART, combination antiretroviral therapy; HBV, hepatitis B virus; anti-HBc, hepatitis B core antibody; HCV, hepatitis C virus; IQR, interquartile ratio; PCV7, 7-valent pneumococcal conjugate vaccine; SD, standard deviation.

### Overall serological responses

**Figure 2:**
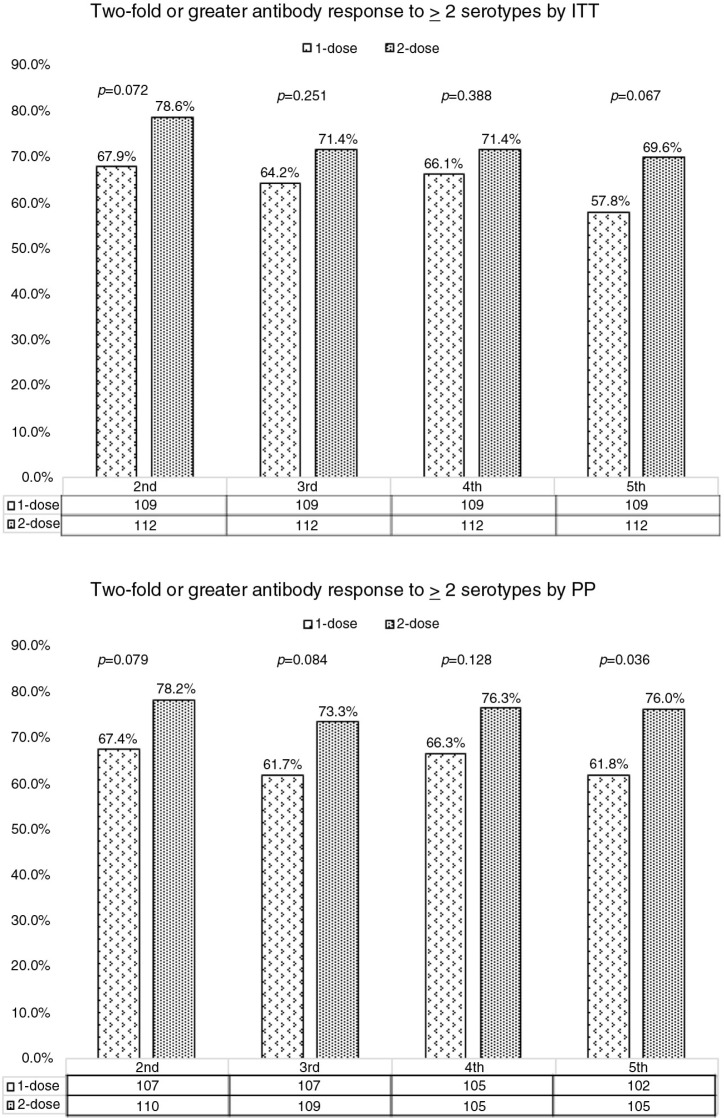
Persistent immune responses defined by a twofold or more immunoglobulin rise to at least two *Streptococcus pneumoniae* serotypes among 221 HIV-positive adult participants in the second, third, fourth and fifth years following vaccination with one or two doses of 7-valent pneumococcal conjugate vaccine by intention-to-treat and per-protocol analyses.

Significant serological responses characterized by a twofold or greater increase in antibody levels to two or more serotypes by ITT and PP analyses are represented in Table 2 and Figure 2. Throughout the five years of follow-up, serological responses were maintained by 57.6 to 67.9% and 69.6 to 78.6% of the vaccinees in the one- and two-dose groups by ITT analysis, respectively. At the end of five years of follow-up, more participants in the two-dose group had persistent serological responses compared to the one-dose group, but this difference was only statistically significant by PP analysis: 68.6 vs 57.8% (*p*=0.067) by ITT and 61.8 vs 76% (*p*=0.026) by PP. Adding the arbitrary cutoff of antibody concentrations ≥1 µg/mL to the primary end point yielded similar results (Table 3).

**Table 2:** Percentage of HIV-positive adults with persistent immune responses defined by a twofold or more IgG rise to at least two *Streptococcus pneumoniae* serotypes in the second, third, fourth and fifth years following one or two doses of PCV7 by intention-to-treat (ITT) and per-protocol (PP) analyses (primary end point)

	One dose	Two doses	*p*
ITT			
Year 2	67.9	78.6	0.072
Year 3	64.2	71.4	0.251
Year 4	66.1	71.4	0.388
Year 5	57.8	69.6	0.067
PP			
Year 2	67.4	78.2	0.079
Year 3	61.7	73.3	0.084
Year 4	66.3	76.3	0.128
Year 5	61.8	76.0	0.026

IgG, immunoglobulin; PCV7, 7-valent pneumococcal conjugate vaccine.

**Table 3:** Percentage of HIV-positive participants with persistent immune responses defined by a twofold or more IgG rise plus an absolute IgG titre >1 µg/mL to at least two *Streptococcus pneumoniae* serotypes in the second, third, fourth and fifth years following one or two doses of PCV7 by intention-to-treat (ITT) and per-protocol (PP) analyses (secondary end point)

	One dose	Two doses	*p*
ITT			
Year 2	67.9	77.7	0.102
Year 3	64.2	70.5	0.317
Year 4	66.1	70.5	0.474
Year 5	57.8	67.9	0.122
PP			
Year 2	69.2	79.1	0.095
Year 3	65.4	72.5	0.262
Year 4	68.6	75.2	0.282
Year 5	61.8	73.8	0.065

IgG, immunoglobulin; PCV7, 7-valent pneumococcal conjugate vaccine.

### Serological responses to individual serotypes

The antibody responses to individual serotypes are shown in Supplementary Figures 1 and 2. Individual response rates were highest to serotype 14, followed by 23F, 6B and 19F. Sequential GMTs of specific anti-capsular IgG antibodies to serotypes 6B, 14, 19F and 23F are shown in Table 4, and the proportions of participants with persistent absolute IgG concentrations >1 µg/mL are shown in Table 5. Both groups maintained increased specific antibody levels above baseline throughout the five years, gradually decreasing thereafter.

**Table 4:** Sequential geometric mean titres (95% confidence interval) of specific anti-capsular immunoglobulin (IgG) antibodies to *Streptococcus pneumoniae* serotypes 6B, 14, 19F and 23F in the second to fifth years following vaccination with one or two doses of PCV7 (secondary end point)

	One dose	Two doses	*p*
Anti-6B serotype IgG levels, µg/mL			
Baseline	0.855 (0.743 to 0.985)	0.774 (0.643 to 0.843)	0.131
Year 2	2.023 (1.780 to 2.300)	1.943 (1.691 to 2.231)	0.670
Year 3	1.896 (2.149 to 1.747)	1.747 (1.537 to 1.987)	0.367
Year 4	1.948 (1.714 to 2.214)	1.760 (1.544 to 2.006)	0.272
Year 5	1.790 (1.571 to 2.040)	1.734 (1.519 to 1.979)	0.734
Anti-14 serotype IgG levels, µg/mL			
Baseline	1.992 (1.656 to 2.397)	1.905 (1.574 to 2.308)	0.739
Year 2	8.493 (7.037 to 10.253)	9.816 (8.182 to 11.776)	0.274
Year 3	7.706 (6.359 to 9.337)	8.696 (7.725 to 10.439)	0.366
Year 4	7.704 (6.355 to 9.338)	8.803 (7.345 to 10.549)	0.317
Year 5	6.980 (5.704 to 8.543)	8.216 (6.760 to 9.985)	0.250
Anti-19F serotype IgG levels, µg/mL			
Baseline	1.917 (1.648 to 2.230)	1.608 (1.354 to 1.910)	0.130
Year 2	3.254 (2.847 to 3.719)	3.265 (2.855 to 3.734)	0.973
Year 3	3.021 (2.649 to 3.446)	2.986 (2.633 to 3.387)	0.899
Year 4	3.277 (2.862 to 3.753)	3.020 (2.646 to 3.446)	0.392
Year 5	3.049 (2.682 to 3.477)	2.892 (2.537 to 3.295)	0.566
Anti-23F serotype IgG levels, µg/mL			
Baseline	0.734 (0.643 to 0.843)	0.644 (0.548 to 0.757)	0.020
Year 2	1.840 (1.554 to 2.180)	1.862 (1.574 to 2.202)	0.924
Year 3	1.709 (1.454 to 2.009)	1.624 (1.385 to 1.904)	0.654
Year 4	1.711 (1.444 to 2.028)	1.567 (1.343 to 1.829)	0.448
Year 5	1.555 (1.315 to 1.839)	1.502 (1.287 to 1.752)	0.760

*P* values compare IgG levels between one- and two-dose groups; IgG, immunoglobulin; PCV7, 7-valent pneumococcal conjugate vaccine.

**Table 5:** Proportions of HIV-positive adults with serotype-specific antibody concentrations of ≥1 µg/mL before and after vaccination with PCV7 between the following two to five years (secondary end point)

	Baseline	Year 2	Year 3	Year 4	Year 5
6B					
One dose	37.6% (41/109)	84.1% (90/107)	86.9% (93/107)	84.8% (89/105)	83.3% (85/102)
Two doses	33.9% (33/112)	82.7% (91/110)	77.1% (84/109)	81.0% (85/105)	79.6% (82/103)
14					
One dose	69.7% (76/109)	100% (107/107)	100% (107/107)	100% (105/105)	100% (102/102)
Two doses	69.6% (78/112)	100% (110/110)	100% (109/109)	100% (105/105)	100% (104/103)
19F					
One dose	81.7% (89/109)	98.1% (105/107)	98.1% (102/107)	97.1% (102/105)	99.0% (101/102)
Two doses	74.1% (83/112)	96.4% (106/110)	95.4% (104/109)	96.2% (101/105)	94.3% (97/103)
23F					
One dose	33.0% (36/109)	74.8% (80/107)	71.0% (76/107)	74.3% (78/105)	67.6% (69/102)
Two doses	30.4% (34/112)	77.3% (85/110)	75.2% (82/109)	75.2% (79/105)	73.8% (76/103)

All *p* values >0.05 when comparing the one-dose and two-dose groups; PCV7, 7-valent pneumococcal conjugate vaccine.

### Factors associated with persistent serological response

Table 6 summarizes the results of linear regression with the GEE approach to define the factors associated with persistent serological response between the second and fifth years of follow-up. Two doses versus one dose of PCV7 (adjusted odds ratio (AOR) 1.71, 95% CI 1.10 to 2.65, *p*=0.016), concurrent cART (OR 2.16, 95% CI 1.16 to 4.00, *p*=0.015) and CD4 lymphocyte recovery (AOR 1.12, per 100 cells/µL gained, 95% CI 1.01 to 1.27, *p*=0.031) were significantly associated with persistent serological responses in the fifth year following vaccination. We repeated the GEE model to incorporate time-updated values for CD4 count and PVL. However, this measure did not change the fact that two doses over one dose and cART were predictive of persistent responses in the fifth year. Time-updated CD4 counts were predictive of persistent responses only in the fifth year (AOR 1.131, 95% CI 1.021 to 1.265, *p*=0.031).

**Table 6:** Adjusted odds ratio (AOR) for persistent significant antibody responses, defined as a twofold or greater increase in specific IgG to two or more serotypes from baseline in the second to fifth years following vaccination with PCV7

	Second year				Third year				Fourth year				Fifth year			
				
	AOR	95% CI		*p*	AOR	95% CI		*p*	AOR	95% CI		*p*	AOR	95% CI		*p*
Age (continuous)	0.986	0.962	1.011	0.259	0.981	0.957	1.006	0.133	0.984	0.961	1.008	0.190	0.984	0.960	1.008	0.195
Two doses vs one dose	**1.786**	**1.123**	**2.840**	**0.014**	**1.623**	**1.027**	**2.566**	**0.038**	**1.604**	**1.024**	**2.513**	**0.039**	**1.711**	**1.104**	**2.652**	**0.016**
On cART vs untreated	1.260	0.621	2.556	0.522	1.763	0.897	3.466	0.100	**1.793**	**0.950**	**3.384**	**0.071**	**2.156**	**1.162**	**4.000**	**0.015**
HBsAg-positive	1.001	0.510	1.965	0.999	1.031	0.528	2.017	0.928	0.896	0.469	1.714	0.741	0.860	0.459	1.611	0.637
Anti-HCV-positive	1.368	0.535	3.497	0.513	1.729	0.647	4.616	0.275	1.602	0.703	3.650	0.262	1.270	0.566	2.849	0.563
Baseline PVL >10^5^ copies/mL	1.909	0.628	5.809	0.255	2.196	0.808	5.970	0.123	1.718	0.673	4.382	0.258	1.713	0.643	4.560	0.281
Time-updated PVL <20 copies/ml	1.022	0.492	2.125	0.954	0.939	0.491	1.796	0.848	0.802	0.421	1.530	0.504	0.766	0.363	1.618	0.485
Nadir CD4 count <200 cells/µL	1.251	0.691	2.266	0.459	1.205	0.669	2.171	0.534	1.268	0.716	2.246	0.416	1.424	0.805	2.518	0.225
Time-updated CD4 counts	1.084	0.937	1.253	0.277	0.993	0.880	1.120	0.908	1.105	0.986	1.239	0.085	**1.131**	**1.012**	**1.265**	**0.031**

cART, combination antiretroviral therapy; CI, confidence interval; HBsAg, hepatitis B surface antigen; HCV, hepatitis C virus; IgG, immunoglobulin; PCV7, 7-valent pneumococcal conjugate vaccine; PVL, plasma HIV RNA load. Bold indicates the variables where *p* is significant <0.05.

### Pneumonia and invasive pneumococcal disease

Fifteen episodes of pneumonia occurred in 11 vaccinated subjects, seven of whom had received one dose of PCV and four of whom had received two doses over a median follow-up duration of 6.5 years (range: 5.8 to 6.8 years) post-vaccination. Only one patient in the one-dose group received a confirmed diagnosis of *S. pneumoniae* pneumonia, 4.2 years after vaccination, on the basis of right lobar pneumonia by chest radiography, sputum smear showing Gram-positive cocci in chains and a positive test for urine pneumococcal antigen. This patient had persistent serological responses to three of four serotypes throughout the five years of follow-up. Two other vaccinees (one in each dosing group) had a probable diagnosis of pneumococcal pneumonia with lobar consolidation and sputum smear showing Gram-positive cocci in chains, sputum cultures yielding “mixed flora” and no other alternative aetiological agent by serological or antigen testing. The patient in the one-dose group was a primary non-responder and the patient in the two-dose group had twofold or greater serological responses to at least two serotypes until the third year but was not tested in the fourth and fifth years of follow-up. No vaccinated subjects had a documented episode of IPD.

### Adverse events

Self-limited injection-related adverse events occurred in 34.3% of our participants, with the most common being injection site soreness (*n*=95). None of the patients who received two doses of PCV reported worsening or new adverse events after receipt of the second dose. There was no statistically significant difference in occurrences of adverse events between patients receiving one or two doses of PCV7 [33].

## Discussion

This study documents durable antibody responses in 58 to 70% of HIV-positive adults who received cART five years after vaccination with PCV7. Our data show that the antibody concentrations post-vaccination remained significantly elevated from baseline and declined very gradually in the subsequent five years, similar to the long-lasting (5- to 10-year) responses elicited against most but not all of the serotypes in children and adolescents post-PCV series in infancy [34–36]. Slow decay of anti-pneumococcal-specific IgG post-PCV in contrast to the more rapid decay post-PPV suggests that the generation of memory B cells via a T-cell-dependent response and natural boosting contributed to antibody persistence [35,37–39]. The levels of antibody persistence are a novel finding for this population, since no prior studies have been conducted in HIV-positive adults with good disease control or in adults with a high incidence of pneumococcal carriage as evidenced by the high prevaccination GMTs with baseline titres greater than 1 µg/mL for two of the four serotypes in our cohort. These high baseline GMTs are in line with the high prevaccination GMTs exceeding 1 µg/mL for all tested serotypes of past or present HIV cohorts in the United States and Spain due to the high pre-PCV incidence of pneumococcal disease and colonization among HIV-positive individuals [40–42]. However, to our knowledge, the present study is the first to examine the long-term (longer than three years) immunogenicity of PCVs in adults, and specifically in those living with HIV (Supplementary Table 1 [24,30,40,43–52]).

Persistent immune responses were more likely to be observed for HIV-positive adults who had received two primary doses administered four weeks apart rather than one dose and among those on cART with CD4 expansion as a surrogate marker for immune reconstitution. In addition, clinical episodes of pneumonia were less frequent for the two-dose than the one-dose group. Only one confirmed case of pneumococcal pneumonia occurred in the one-dose group, and no cases of IPD occurred in the vaccinated cohort in the follow-up period of 6.5 years. Hence, perhaps with a larger sample size, the statistical trend of 57.8 vs 68.6% (*p*=0.067) by ITT analysis between the single- and double-dosing strategies will prove to be a real difference.

Although long-term data for HIV-positive adults receiving PCVs are not available for comparison, there are a few long-term (4- to 5-year) immunogenicity studies of HIV-positive children [53,54] and midterm (1.5- to 3-year) studies of HIV-negative adults [51,55,56]. Of HIV-positive children who received three doses of 9-valent PCV at infancy, 36 to 77% harboured persistent immune responses (defined as ≥0.35 µg/mL of serotype-specific antibody) at five years against serotypes 6B, 14, 19F and 23F [54]. Of older (aged 2 to 18 years) HIV-positive children who received two doses of PCV7 plus one dose of PPV23, 82% had persistent immune responses (≥0.5 µg/mL) at five years against serotypes 6B and 14 [53]. Of our cohort, 68 to 100% had persistent immune responses (using the threshold of 1 µg/mL adopted for adults as a measure of seroprotection [9,57]) at five years against serotypes 6B, 14, 19F and 23F. The percentage of persistent responders (≥1 µg/mL) to the two vaccine serotypes with generally the lowest responses (6B and 23F) for rheumatoid participants after one dose at 1.5 years was remarkably lower (21%) than our one-dose HIV-positive vaccinees (72%) at two years [51].

Despite the different definitions for durability, serotype-differences in durability were similar across diverse populations. The most durable response following PCV7 was observed for serotype 14 in our HIV-positive adults at five years, adults with chronic pulmonary obstructive disease at two years and adults post-renal transplantation at three years [56,58]. Similarly, the greatest decay in serotype-specific antibody was observed for serotype 23F for HIV-positive children and adults with rheumatic diseases, as well as our HIV-positive adults [51,53,54]. However, this does not predict an increased risk of IPD by serotype 23F. A recent study showed serotype-specific correlates of protection in healthy children were lower than 0.35 µg/mL for serotypes 6B and 23F and higher than 0.35 µg/mL for 19F, supporting the notion that there may be variability in the threshold of antibody concentrations required for protection against invasive disease for different serotypes [59]. An epidemiological survey from 2000 to 2012 showed that serotypes 14 and 23F were the two most common serotypes causing IPD in adults in Taiwan [33].

For this reason, and to adjust for the high prevaccination concentrations of antibodies to common serotypes among adults with many years of exposure to pneumococci [58], the primary end point in our study used only the fold increase in antibody concentrations to denote immunogenicity. Using a twofold increase in IgG titres, only 13 to 39% of adult renal transplant recipients compared to 40 to 78% of our HIV-positive participants maintained responses three years after one dose of PCV7 [56].

Double dosing (1 ml) or multiple sequential doses have been administered to improve primary immune responses and also to extend durability of these responses [49,52,60,61]. Previously, we demonstrated superiority of two doses of PCV7 over one dose for HIV-positive adults up to 48 weeks post-vaccination [22]. Here, we show that the dose-response persists after five years. However, in the only clinical efficacy trial of PCV7 in HIV-positive adults receiving two doses one month apart (matching dosing schedule), vaccine efficacy dropped dramatically from 85 to 25% after the first year [23]. Hence, two doses of PCV7 may not be sufficient to prevent recurrent IPD in the less immunocompetent (13% cART coverage and median CD4 212 cells/µL), more at risk (recent IPD on average 19 days earlier) HIV-positive participants in the African study compared to our participants. Yet, a study of HIV-treated adults given three doses of PCV13 administered at six-month intervals failed to demonstrate the value added by the second and third doses in terms of geometric fold rises [61]. The lack of dose-response may be related to the fact that PCV13 was being used as booster vaccinations in individuals previously vaccinated with PPV23 in the study by Glesby and colleagues and not in vaccine-naïve subjects as in our study. Moreover in the PCV13 study, antibody responses beyond one month after each vaccination were not evaluated and, therefore, the long-lasting value of multiple doses could not be evaluated. Current guidelines recommending only a single dose of PCV for HIV-positive adults do not take into account the durability of antibody responses due to the lack of long-term data [11,41].

Like the long-term studies of HIV-positive children, our data show a significant positive association between persistent antibody responses, receipt of cART and duration of cART [53]. In our multivariate analysis, we show that receipt of cART becomes a significant predictor of significant immune responses in the fourth and fifth years but not in the second and third years; that is, cART takes time to have an effect. This finding is consistent with studies showing persistent defects in pneumococcal antigen specific immunity by IFN-gamma ELISpot, T-cell proliferation, CD154 expression and intracellular cytokine assays despite 12 months of cART and persistently higher *S. pneumoniae* carriage rates despite 18 months of cART [6,8]. As the extent of immune recovery at 12 months was greater than at three or six months after cART, the capacity for ongoing reconstitution over time and subsequent effect thereof on immunogenicity and induced-immunological memory of PCV is implied [8]. The late effects of cART could also be inferred from the similar responses found for immunologically AIDS patients immunized immediately compared to those who had received cART for 6 to 12 months before vaccination [62]. The lack of benefit from delaying vaccination and our findings showing that baseline CD4 counts and PVL were not predictive of long-lasting immune responses support the current recommendations of vaccinating all individuals at the time of HIV diagnosis [12,14].

Widespread PCV vaccination of children contributing to herd immunity may decrease the burden of pneumococcal disease in adults [39,63]. However, given the high pre-PCV incidence of IPD in the HIV-positive population, herd effect is unlikely to negate the importance of targeted PCV vaccination [63]. In addition, the incidence of IPD among HIV-positive injecting drug users remains unchanged even in the post-PCV, post-HAART era [64]. For the underprivileged individuals and communities bereft of the benefits of pneumococcal childhood immunization, the role of targeted PCV vaccination of HIV-positive adults continues to be highly relevant.

The 13-valent PCV (PCV13, Prevnar 13), licensed by the US Food and Drug Administration in 2010 for prevention of IPD and otitis media among young children, has now superseded PCV7 [65]. PCV13 contains the seven serotypes included in PCV7 (serotypes 4, 6B, 9V, 14, 18C, 19F and 23F) and six additional serotypes (serotypes 1, 3, 5, 6A, 7F and 19A). PCV13 has comparable immunogenicity to the serotypes common with PCV7 and a comparable adverse reaction profile to PCV7 [66]. Hence, it may be possible to extrapolate the findings of our study to PCV13.

Data on the PCVs are too preliminary to suggest the optimal timing of booster vaccination in adults [43–48,50,57]. Our study addresses this gap, although it was not designed to answer questions on dosing schedules. Given cART, the implications for HIV-positive adults receiving a single or a double 0.5-ml dose of PCV are that they are less likely to require a booster after five years than HIV-positive adults receiving PPV initially. In our study, although two doses were associated with persistent immune responses in the fifth year, in terms of cost-effectiveness, one dose may be sufficient given cART.

There are several limitations to be acknowledged. First, our study was not a randomized trial and no subjects received placebo; however, we performed matched pair analyses to minimize potential confounding factors. Second, we did not compare serological responses to vaccination with PCV vs PPV; further studies are needed to compare the long-term benefits of boosting with PCV or PPV. Third, we did not perform opsonophagocytic assays (OPAs) since OPA titres ≥8 were not necessarily predictive of IPD in children [59] and OPA results appear to correlate well with antibody concentrations even in immunocompromised hosts [54,67]. Fourth, our findings may not apply to women. Lastly, a larger sample size may be necessary to render the long-term dose-effect statistically significant.

We conclude that primary vaccination with one or two 0.5-ml doses of PCV7 achieved durable serological responses in HIV-treated adults throughout the five years of follow-up. This remarkable persistence of antibody responses in contrast to the more rapid decline seen in other immunocompromised populations is sustained by long-term antiretroviral therapy and immune reconstitution.

## Supplementary Material

Long-term immune responses and comparative effectiveness of one or two doses of 7-valent pneumococcal conjugate vaccine (PCV7) in HIV-positive adults in the era of combination antiretroviral therapy
