# Supplementary material for: Long-term immune responses and comparative effectiveness of one or two doses of 7-valent pneumococcal conjugate vaccine (PCV7) in HIV-positive adults in the era of combination antiretroviral therapy
Source: J Int AIDS Soc. 2016 Jan 29;19(1):20631. doi: 10.7448/IAS.19.1.20631 (PMC4733944; doi:10.7448/IAS.19.1.20631)
Supplement: Long-term immune responses and comparative effectiveness of one or two doses of 7-valent pneumococcal conjugate vaccine (PCV7) in HIV-positive adults in the era of combination antiretroviral therapy [file JIAS-19-20631-s001.pdf]

Supplementary Figure 1. Persistent immune responses defined by a 2-fold or more IgG rise to individual pneumococcal serotypes (6B, 14, 19F, and 23F) among 221 HIV-positive adult participants in the 2nd, 3rd, 4th and 5th years following vaccination with one or two doses of PCV7 by intention-to-treat (ITT) and per-protocol (PP) analyses.

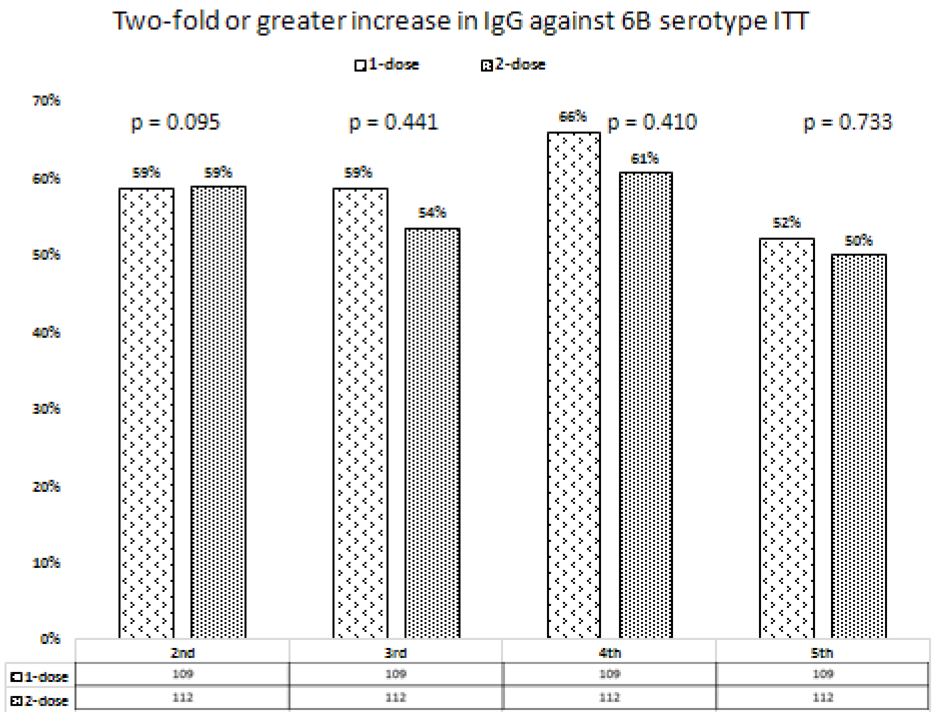

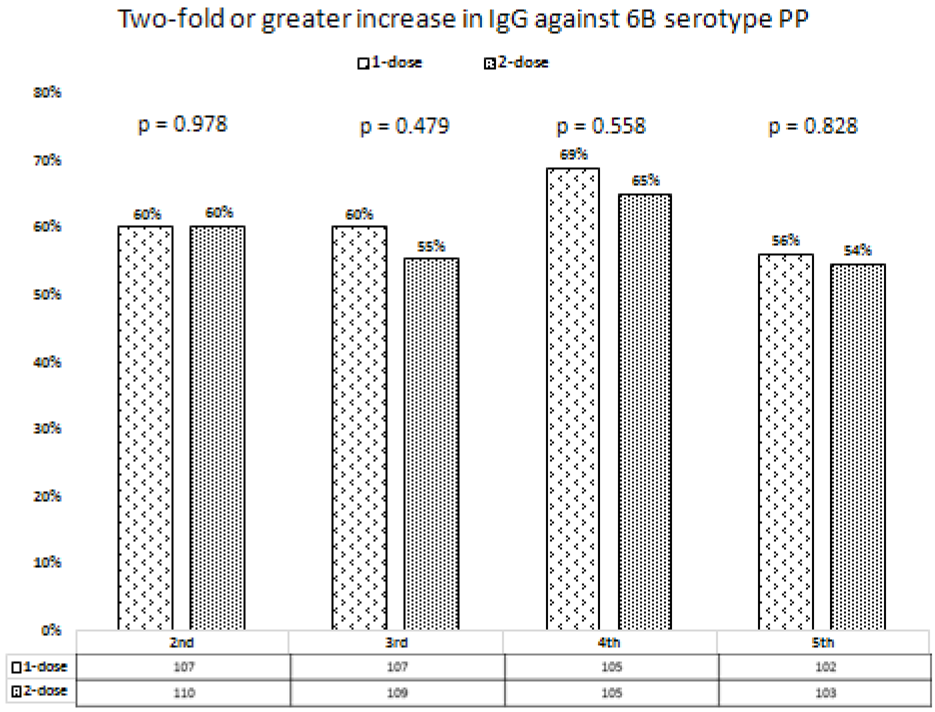

Two-fold or greater increase in IgG against 14 serotype ITT

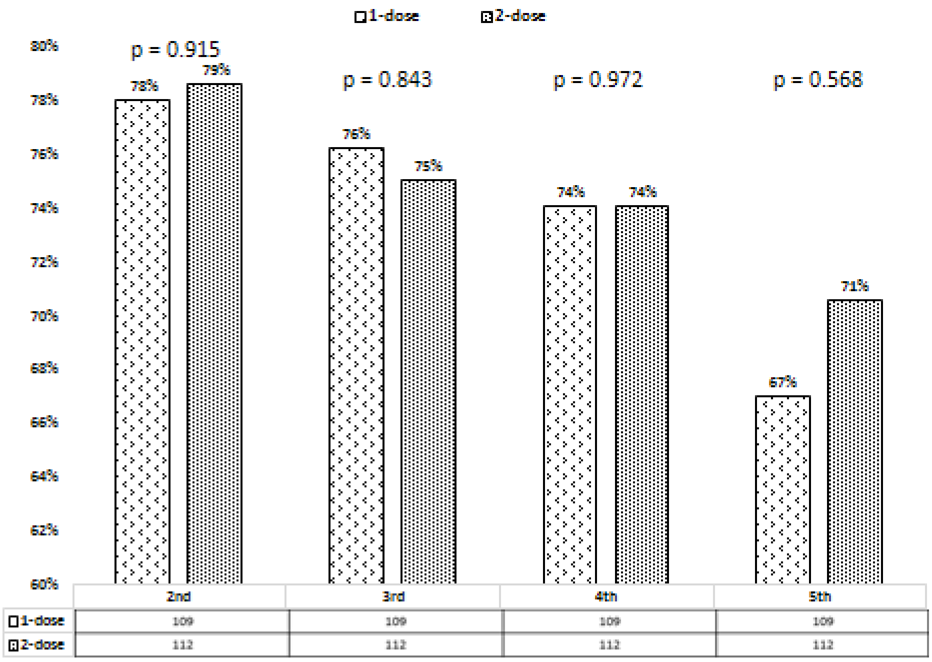

Two-fold or greater increase in IgG against 14 serotype PP

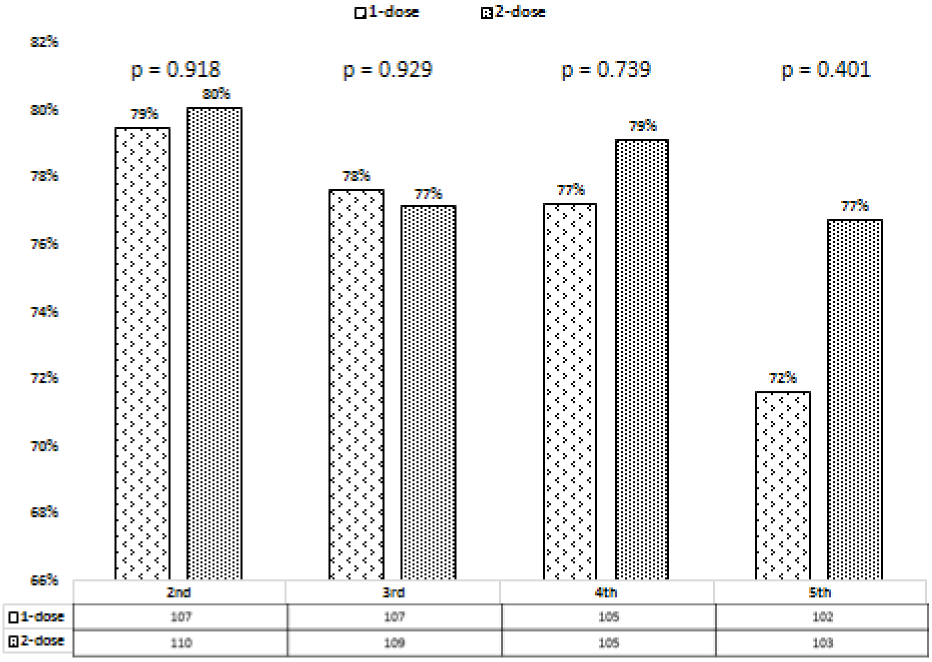

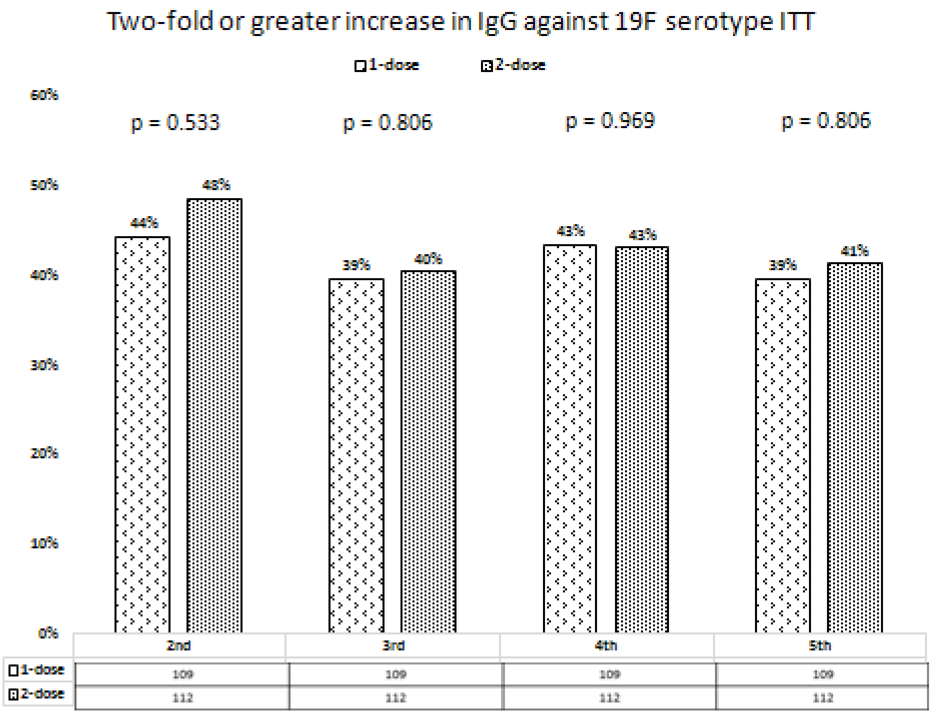
Two-fold or greater increase in IgG against 19F serotype ITT
1-dose
2-dose
60%
50%
40%
30%
20%
10%
0%
p = 0.533
p = 0.806
p = 0.969
p = 0.806
44%
48%
39%
40%
43%
43%
39%
41%
2nd
3rd
4th
5th
1-dose 109 109 109 109
2-dose 112 112 112 112

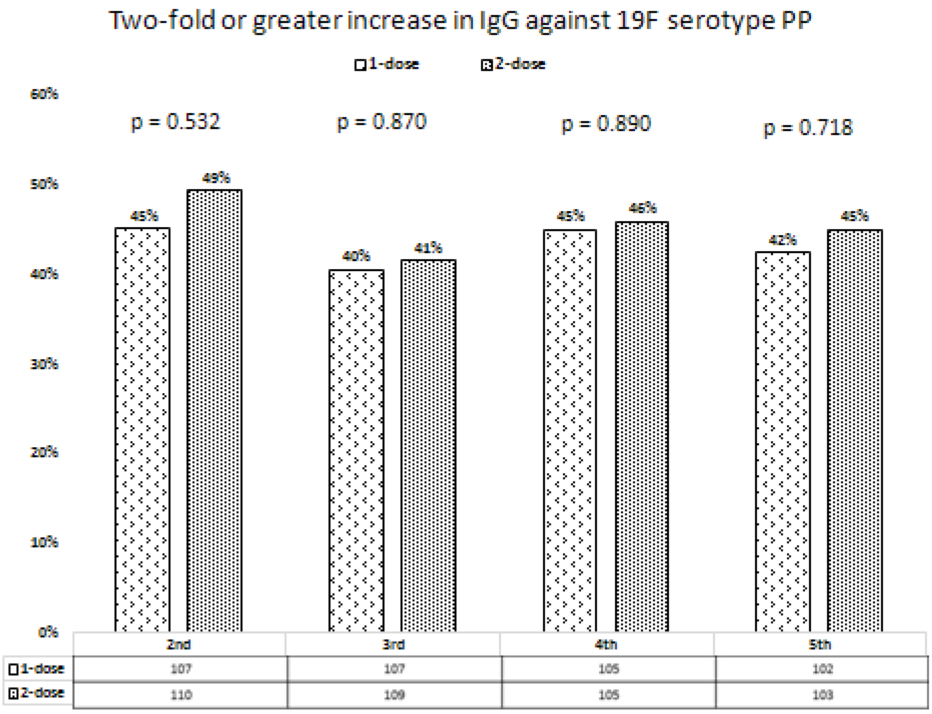
Two-fold or greater increase in IgG against 19F serotype PP
1-dose
2-dose
60%
50%
40%
30%
20%
10%
0%
p = 0.532
p = 0.870
p = 0.890
p = 0.718
45%
49%
40%
41%
45%
46%
42%
45%
2nd
3rd
4th
5th
1-dose 107 107 105 102
2-dose 110 109 105 103

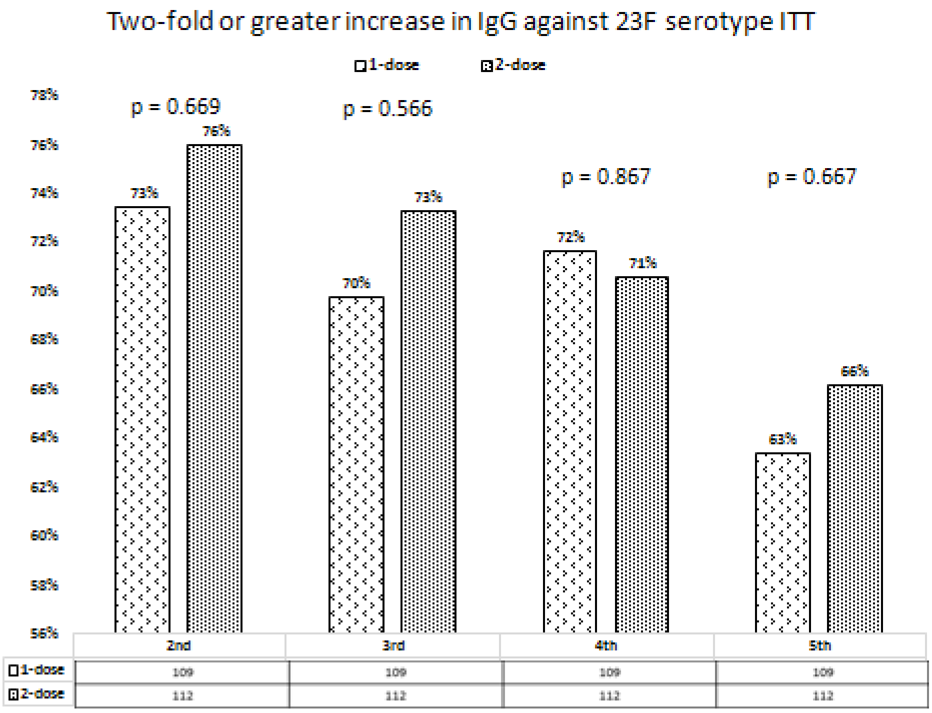
Two-fold or greater increase in IgG against 23F serotype ITT
1-dose
2-dose
78%
76%
74%
72%
70%
68%
66%
64%
62%
60%
58%
56%
p = 0.669
p = 0.566
p = 0.867
p = 0.667
73%
76%
70%
73%
72%
71%
63%
66%
2nd
3rd
4th
5th
1-dose 109 109 109 109
2-dose 112 112 112 112

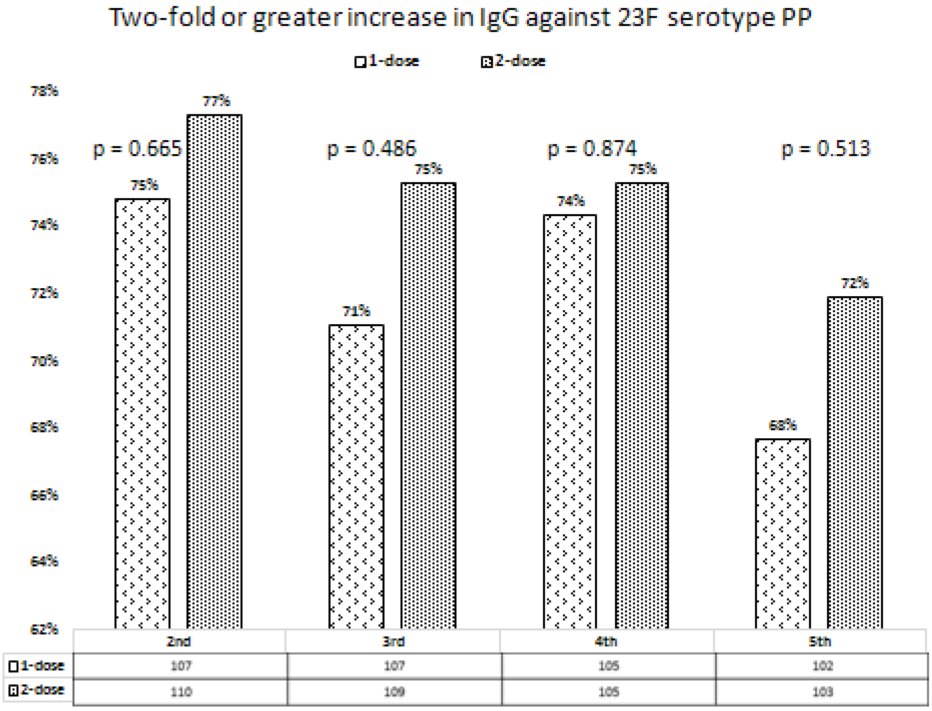
Two-fold or greater increase in IgG against 23F serotype PP
1-dose
2-dose
78%
76%
74%
72%
70%
68%
66%
64%
62%
p = 0.665
p = 0.486
p = 0.874
p = 0.513
75%
77%
71%
75%
74%
75%
68%
72%
2nd
3rd
4th
5th
1-dose 107 107 105 102
2-dose 110 109 105 103

Supplementary Figure 2. Box plots of sequential geometric mean titers (95% confidence interval) of specific anti-capsular immunoglobulin (IgG) antibodies to *Streptococcus pneumoniae* serotypes 6B, 14, 19F, and 23F in the 2nd to 5th years following vaccination with one or two doses of PCV7 (secondary endpoint).

**1 Dose**	**2 Doses**
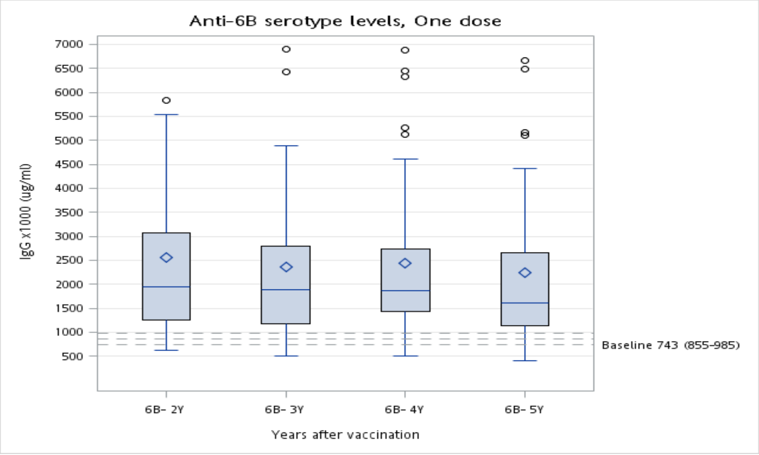	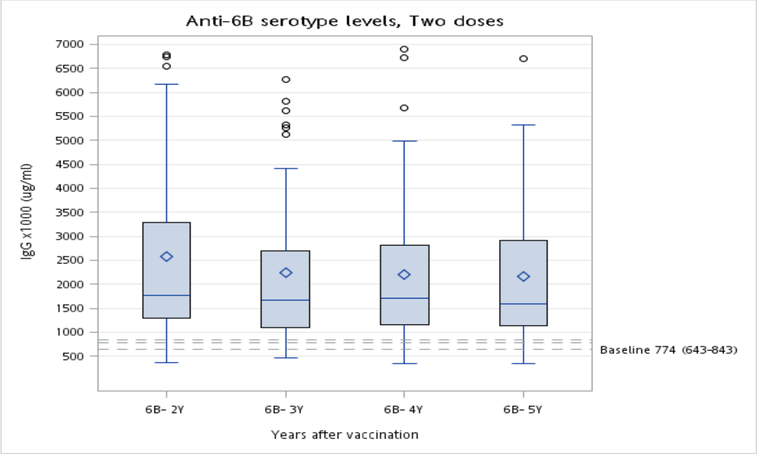

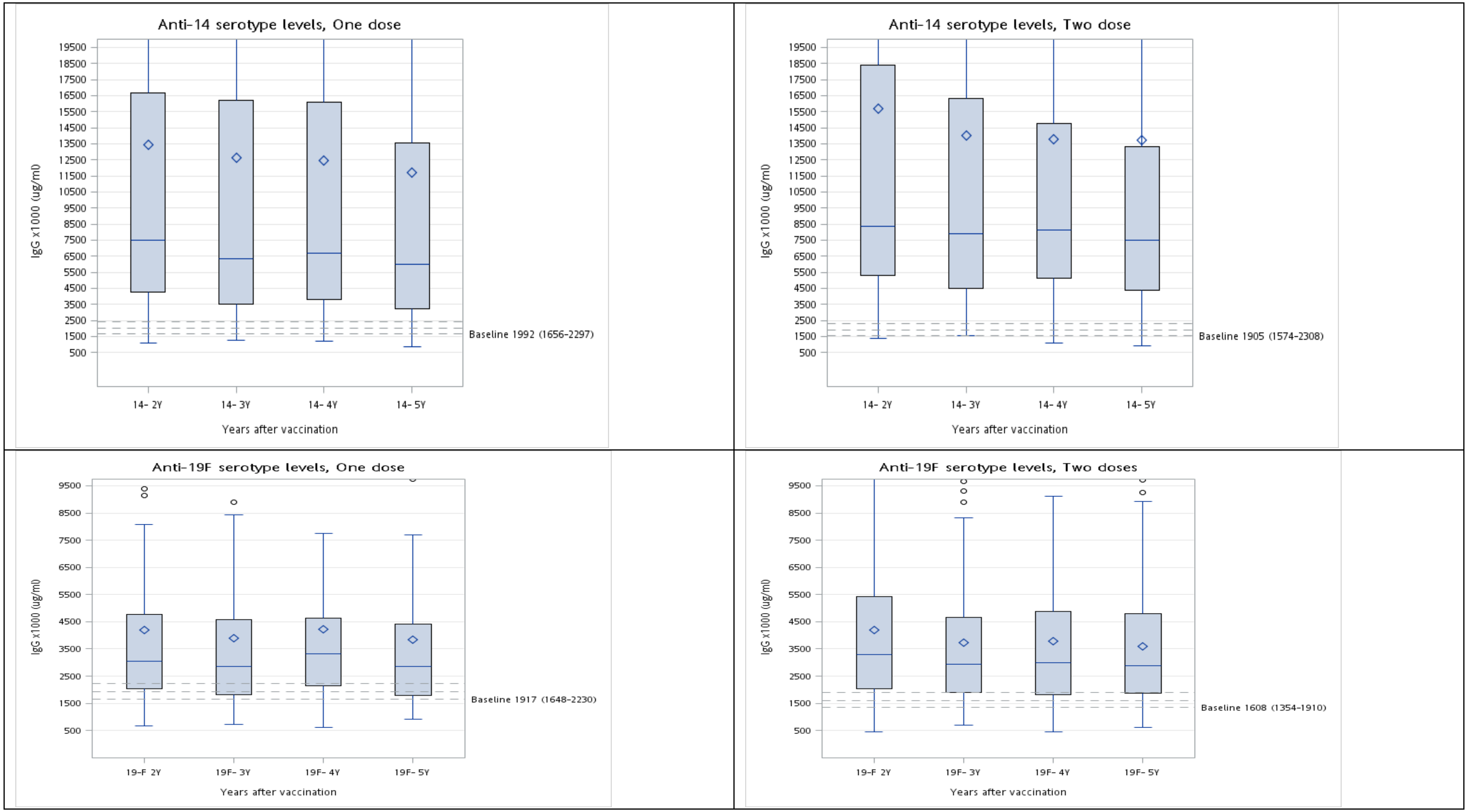

Anti-14 serotype levels, One dose
IgG x1000 (ug/ml)
Baseline 1992 (1656-2297)
14- 2Y
14- 3Y
14- 4Y
14- 5Y
Years after vaccination
Anti-14 serotype levels, Two dose
IgG x1000 (ug/ml)
Baseline 1905 (1574-2308)
14- 2Y
14- 3Y
14- 4Y
14- 5Y
Years after vaccination
Anti-19F serotype levels, One dose
IgG x1000 (ug/ml)
Baseline 1917 (1648-2230)
19-F 2Y
19F- 3Y
19F- 4Y
19F- 5Y
Years after vaccination
Anti-19F serotype levels, Two doses
IgG x1000 (ug/ml)
Baseline 1608 (1354-1910)
19-F 2Y
19F- 3Y
19F- 4Y
19F- 5Y
Years after vaccination

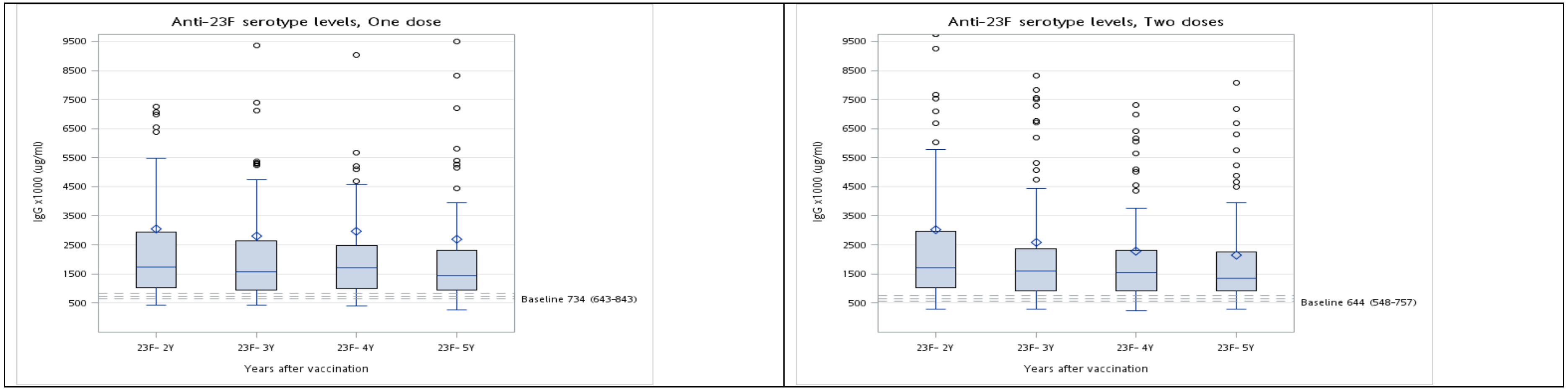

Note: The length of the box represents the interquartile range (the distance between the 25th and 75th percentiles).

- The symbol in the box interior represents the group mean.
- The horizontal line in the box interior represents the group median.
- The vertical lines (called whiskers) issuing from the box extend to the group minimum and maximum values

Supplementary Table 1. Comparative studies of immunocompromised adults with specific anti-pneumococcal capsular IgG geometric mean titers (GMTs) assayed before and following PCV7 vaccination ordered from shortest to longest duration of serological follow-up.

Reference [no.] (Year) Country Population	Follow-up after last PCV, vaccination schedule	Timing of immune assays	No. of subjects	6B	14	23F	Response definition	Response Rate (%)
Feikin [40] (2001) USA, HIV adults	2 months (PCV7/PCV7) at 0, 2 months	0, 2, 4 mo.	PCV7/PCV7<br>N = 15	Pre- 0.99<br>Post 2 mo- 1.7<br>Post 4 mo- 2.0	Pre- 5.0<br>Post 2 mo- 9.8<br>Post 4 mo- 10.3	Pre- 0.77<br>Post 2 mo- 1.4<br>Post 4 mo- 1.5	>2 fold increase	4 mo:<br>40% (6B)<br>28% (14)<br>50% (23F)
	(PCV7/PPV23) at 0, 2 months		PCV7/PPV<br>N = 18	Pre- 1.2<br>Post 2 mo- 2.0<br>Post 4 mo- 2.2	Pre- 5.0<br>Post 2 mo- 10.7<br>Post 4 mo- 11.4	Pre- 1.2<br>Post 2 mo- 2.0<br>Post 4 mo- 1.8		44% (6B)<br>40% (14)<br>46% (23F)
Ho [43] (2013) Brazil, HIV adults	6 months (PCV7/PLA) at 0, 2 months	0, 2, 6, mo.	PCV7/PLA<br>N = 110	Pre- 0.51<br>Post 6 mo- 1.24	Pre- 1.15<br>Post 6 mo- 9.32	-	GMT ≥1μg /ml	6 mo:<br>49.5% (6B)<br>83.5% (14)
	(PCV7/PPV23) at 0, 2 months		PCV7/PPV<br>N = 110	Pre- 0.57<br>Post 6 mo- 1.76	Pre- 1.40<br>Post 6 mo- 6.35	-		6 mo:<br>59.3% (6B)<br>84.6% (14)
Gordon [44] (2007) Malawi, HIV adults	6 months PCV at 0 month	0, 1, 6, mo.	PCV<br>N = 10	Pre- 0.25<br>Post 1 mo- 1.89<br>Post 6 mo- 2.6	Pre- 0.71<br>Post 1 mo- 18.9<br>Post 6 mo- 6.6	Pre-0.49<br>Post 1 mo- 8.9<br>Post 6 mo- 4.8	-	-
Crum-Cianflone [30] (2010) USA, HIV adults	6 months (PCV7) one dose in PPV experienced	0, 0.5, 2, 6 mo.	PCV<br>N = 131	-	Pre- 0.86<br>Post 6 mo- 3.0	-	>2 fold increase plus ≥1 μg/ml	6mo:<br>41% (14)<br>41% (2/4 serotypes)
Lesprit [24] (2007) France, HIV adults	6 months (PCV7/PPV23) at 0, 1 month	0, 1, 2, 6 mo.	PCV7/PPV<br>N = 105	Pre- 0.47<br>Post 6 mo- 1.46	Pre- 1.68<br>Post 6 mo – 5.82	Pre- 0.24<br>Post 6 mo- 1.26	>2 fold increase plus ≥1 μg/ml	6mo:<br>84% (≥1 serotype)
Deloria-Knoll, [45] (2006) USA, HIV adults	7 months (PCV7/PPV23) at 0, 6 months	0, 1, 6, 7, mo.	PCV7/PPV<br>N = 118	Pre- 1.47<br>Post 7 mo – 4.75	Pre- 1.67<br>Post 7 mo- 8.3	Pre- 0.84<br>Post 7 mo-3.4	-	-

**Sogaard [46] (2010) Denmark, HIV adults**	**7 months (PCV/PCV/PPV23) at 0, 3, 9 months**	**0, 3, 4, 9, 10 mo**	**No TLR9 adjuvant N = 49**	**Pre- 1.23 Post 9 mo- 2.62 Post 10 mo- 4.07**	**Pre- 2.38 Post 9 mo- 7.64 Post 10 mo- 10.1**	**Pre- 0.75 Post 9 mo- 2.98 Post 10 mo- 4.09**	**2-fold increase plus ≥1 μg/mL**	**10mo: 51.1% (5/7 serotypes)**
			**TLR9 agonist N = 48**	**Pre- 1.03 Post 9 mo – 3.55 Post 10 mo – 5.21**	**Pre- 1.92 Post 9 mo- 7.31 Post 10 mo- 9.76**	**Pre-0.69 Post 9 mo- 3.36 Post 10 mo- 5.14**		**10mo: 87.8% (5/7 serotypes)**
**Chen [47] (2008) Uganda, HIV adults**	**8 months (PCV7/ PPV23) at 0, 2 months**	**0, 2, 3, 8 mo**	**CD4 200-499 N = 30**	**-**	**Pre- 5.27 Post 8 mo- 17.73**	**-**	**GMT ≥0.35 μg /ml**	**2 mo: 100% (14)**
			**CD4 ≥500 N = 28**	**-**	**Pre- 5.10 Post 8 mo- 28.66**	**-**		**2 mo: 100% (14)**
**Musher [48] (2008) USA, Veterans post-pneumococcal pneumonia**	**12 months, (PCV7/PPV23) (PPV23/PCV7) at 0, 6 months in PPV before**	**0, 2, 6, 8, 12 mo**	**PCV7/PPV N = 37**	**Pre- 0.65 Post 6 mo- 1.21 Post 12 mo- 0.88**	**Pre- 2.23 Post 6 mo- 5.66 Post 12 mo- 3.99**	**Pre- 0.44 Post 6 mo- 2.27 Post 12 mo- 1.25**	**-**	**-**
**Jackson [49], (2007) USA, Seniors 70-79 years**	**13 months (PCV7/PPV23) 0, 12 months PPV before**	**0, 1, 12, 13 months**	**0.5-ml dose N = 50**	**Pre- 1.0 Post 12 mo- 1.2 Post 13 mo- 1.7**	**Pre- 4.0 Post 12 mo- 6.5 Post 13 mo- 7.7**	**Pre- 0.7 Post 12 mo- 1.5 Post 13 mo- 1.9**	**-**	**-**
			**1.0-ml dose N= 50**	**Pre- 1.1 Post 12 mo- 1.8 Post 13 mo- 2.3**	**Pre- 2.4 Post 12 mo- 5.1 Post 13 mo- 5.8**	**Pre- 0.8 Post 12 mo- 2.8 Post 13 mo- 3.3**	**-**	**-**
**Tobudic [50] (2012) Austria, Renal transplant**	**14 months (PCV/PPV) at 0, 12 months**	**0, 2, 12, 14 mo**	**(PCV7/PPV) N = 33**	**Pre- 2.13 Post 12 mo- 3.38 Post 14 mo- 8.11**	**-**	**-**		
**Crnkic Kapetanovic, [51] (2013) Sweden,**	**18 months (PCV) at 0 month**	**0, 1, 18 mo**	**Rheumatoid arthritis N = 163**	**Pre- 1.4-2.0 Post 18 mo- 0.6-1.2**	**-**	**Pre- 0.7-0.97 Post 18 mo- 0.8-2.0**	**GMT ≥1μg/ml**	**18-40% (2 serotypes)**

**Arthritis adults**								
			**Spondylo-arthropathy<br>N = 139**	**Pre- 1.5-2.9<br>Post 18 mo- 1.0-2.8**	**-**			**48-70% (2 serotypes)**
**Dransfield [52] (2012) USA, COPD adults, 63 years**	**24 months (PCV) at 0 months 51% had PPV before**	**0, 1, 12, 24 mo**	**1.0ml dose<br>N = 91**	**Pre- 0.9<br>Post 12 mo- 1.77<br>Post 24 mo- 1.33**	**Pre- 2.53<br>Post 12 mo- 7.47<br>Post 24 mo- 5.68**	**Pre- 0.74<br>Post 12 mo- 2.73<br>Post 24 mo- 1.99**	**-**	**-**
**Present Study (2015)<br>Taiwan,<br>HIV adults**	**5 years<br>(PCV) at 0 mo. or 0, 1 month**	**0, 24, 36, 48, 60 mo**	**0.5-ml dose<br>N = 109**	**Pre- 0.86<br>Post 24 mo- 2.02<br>Post 60 mo- 1.79**	**Pre- 1.99<br>Post 24 mo- 8.49<br>Post 60 mo- 6.98**	**Pre- 0.73<br>Post 24 mo- 1.84<br>Post 60 mo- 1.56**	**2-fold increase**	**57.8% (2 serotypes)**
			**0.5-ml dose x 2<br>N = 112**	**Pre- 0.77<br>Post 24 mo- 1.94<br>Post 60 mo- 1.73**	**Pre- 1.91<br>Post 24 mo- 9.82<br>Post 60 mo- 8.22**	**Pre- 0.64<br>Post 24 mo- 1.86<br>Post 60 mo- 1.50**		**69.6% (2 serotypes)**
